# Distribution of Silicified Microstructures, Regulation of Cinnamyl Alcohol Dehydrogenase and Lodging Resistance in Silicon and Paclobutrazol Mediated *Oryza sativa*

**DOI:** 10.3389/fphys.2017.00491

**Published:** 2017-07-12

**Authors:** Deivaseeno Dorairaj, Mohd Razi Ismail

**Affiliations:** ^1^Department of Crop Science, Faculty of Agriculture, Universiti Putra Malaysia Serdang, Malaysia; ^2^Laboratory of Climate-Smart Food Crop Production, Institute of Tropical Agriculture and Food Security, Universiti Putra Malaysia Serdang, Malaysia

**Keywords:** CAD, lignin, lodging, rice, SEM-EDX, silicon

## Abstract

Lodging is a phenomenon that affects most of the cereal crops including rice, *Oryza sativa*. This is due to the fragile nature of herbaceous plants whose stems are non-woody, thus affecting its ability to grow upright. Silicon (Si), a beneficial nutrient is often used to toughen and protect plants from biotic and abiotic stresses. Deposition of Si in plant tissues enhances the rigidity and stiffness of the plant as a whole. Silicified cells provide the much needed strength to the culm to resist breaking. Lignin plays important roles in cell wall structural integrity, stem strength, transport, mechanical support, and plant pathogen defense. The aim of this study is to resolve effects of Si on formation of microstructure and regulation of cinnamyl alcohol dehydrogenase (CAD), a key gene responsible for lignin biosynthesis. Besides evaluating silicon, paclobutrazol (PBZ) a plant growth retartdant that reduces internode elongation is also incorporated in this study. Hardness, brittleness and stiffness were improved in presence of silicon thus reducing lodging. Scanning electron micrographs with the aid of energy dispersive x-ray (EDX) was used to map silicon distribution. Presence of trichomes, silica cells, and silica bodies were detected in silicon treated plants. Transcripts of CAD gene was also upregulated in these plants. Besides, phloroglucinol staining showed presence of lignified vascular bundles and sclerenchyma band. In conclusion, silicon treated rice plants showed an increase in lignin content, silicon content, and formation of silicified microstructures.

## Introduction

Rice, the main source of carbohydrate for about half the human race is cultivated globally from tropical and temperate Asia, Europe, Africa, North and South America to Australia in a variety of climatic conditions ranging from wettest area to the driest deserts. It can be grown in rainfed lowlands, rainfed uplands, mangroves, and even in deepwater areas such as Irrawaddy Delta in Thailand and Mekong in Vietnam and Cambodia. However, the lowland rice agriculture is the primary way of cultivation as it is now responsible for 86% of the total world rice crop and the yields are typically in the range of 2.0–3.5 t ha^−1^ (Ladha et al., 1997). Though rice plant is highly adaptive, its production is not parallel to population increase in most of the rice producing and consuming countries. To meet the ever increasing demand for this crop, yield has to be increased many folds without compromising its quality.

Lodging is defined as permanent displacement of stem from its upright position (Fageria et al., 2006). It mostly occurs just before harvest when the plant in particular the lower portion of the stem is unable to withstand the weight of panicle. To date, three types of lodging had been recorded, namely bending of stems at the base, breakage of stems at any point along the length and root lodging (Gowariker et al., 2009). The former depends on the tensile failure strength of the first internodes, as well as on stem wall diameter and thickness (Verma et al., 2005). The second type of lodging occurs as a result of excessive bending pressure at the higher internodes, and is determined primarily by the morphology and quality of the culm (Islam et al., 2007). Meanwhile, the latter ensues in response to loose and shallow roots with poor anchorage.

Often considered as an anomaly, silicon (Si) accumulation sometimes exceeds that of essential plant nutrients especially in the grass family (Epstein, 1994). It is absorbed as silicic acid and make up to 10% of dry weight of monocot plants such as rice (Epstein, 1994). Si is primarily present in the epidermal cells which provide structural rigidity to the plant. A noteworthy unique feature of silicon is that once it is deposited as silica gel, it is not re-distributed to other parts of the plant (Rodrigues et al., 2005). Thus, application of Si should be done at the right time to ensure plants realize its potential benefit.

However, the function and application of Si is often neglected due to its classification as a non-essential element. It helps alleviate both biotic and abiotic stresses (Ma, 2004) besides improving crop productivity. The latter is possible as Si in rice plants can increase photosynthesis and decrease susceptibility to disease and insect damage as well as prevent lodging. Since grain shattering and percentage of unfilled grains are reduced, yield and yield components fare well in Si-fertilized fields.

Lignin which is derived from three monolignols of *p*-coumaryl, coniferyl, and sinapyl alcohols is the second major biopolymer component of the plant cell wall after cellulose which provides rigidity (Boerjan et al., 2003). These precursors of lignin are synthesized *via* phenylpropanoid pathway. Many enzymes and their corresponding genes involved in this pathway have been characterized and cloned (Boudet et al., 1995; Whetten and Sederoff, 1995). The last step of the monolignol pathway is the reduction of cinnamaldehydes to cinnamyl alcohols whereby it is catalyzed by cinnamyl alcohol dehydrogenase (CAD), which plays crucial role in the production of lignin monomers, and an increased expression of this enzyme could indicate increased lignification (Walter et al., 1988). The relative proportions of these cinnamyl alcohols are important factors that determine the structural and mechanical properties of lignin (Sibout et al., 2003).

In plants, lignin, a complex phenolic polymer is mainly deposited in secondary thickened cell walls by cross-linking with cellulose and hemicellulose enhancing its rigidity. This provides structural support to the wall and assists in the transport of water and nutrients within xylem tissue by decreasing the permeability of the cell wall (Boerjan et al., 2003). In addition, the insolubility and complexity of the lignin polymer makes it resistant to degradation by most microorganisms (Brill et al., 1999; Chabannes et al., 2001; Jones et al., 2001).

A brittle culm is often the product of a compromised physical strength which is determined by composition of plant cell wall. This trait is of paramount interest in cereal crops as weak stem strength will lead to a lodging phenotype (Hai et al., 2005). Ma (2010) studied the expression of TaCAD1 (CAD in *Triticum aestivum*) in two wheat varieties, namely H4564 and C6001 which were lodging resistant and lodging susceptible, respectively. The lodging resistant variety had a lower lodging index and higher lignin content than its counterpart, all of which attributed to a stronger stem (Ma, 2009). CAD transcripts in stems were highly expressed at the elongation and heading stages in both varieties (Ma, 2010). Conversely, RNA transcripts of CAD at milky stage showed contrasting results whereby it was high in H4564 but declined in the lodging susceptible variety (Ma, 2010). This is an interesting and valid outcome as milky stage is the time when plants are most prone to lodging. Results of TaCAD1 mRNA abundance, protein content, and enzyme assay were correlated to lignin contents and lodging indices of the two wheat varieties studied (Ma, 2010). It is postulated that a high expression of CAD would be supported by an increase in lignin synthesis which would then enhance the strength and lodging resistance of stems (Ma, 2010).

Likewise, higher silicon contents are also related to physical strength (Ma and Yamaji, 2006). It is noteworthy to state that on a unit weight basis, the energetic cost of incorporating silica is only 3.7 and 6.7% to that of incorporating lignin and cell-wall carbohydrate according to Raven (1983). This proves that besides being an energetically inexpensive structural component of cell walls, its erect habit and the disposition of the leaves of plants amply supplied with Si favors light interception and, hence, photosynthesis (Epstein, 1994).

Plant growth regulators are known to control many processes within plants. There are various reports on the effect of exogenously applied plant growth regulators on plant growth and development (Bevilaqua et al., 1993, 1995; Pan and Zhao, 1994; Asborno et al., 1999). Triazoles are a component of plant growth regulators of which paclobutrazol (PBZ) is a member. They inhibit gibberellins biosynthesis and as such prominently known as plant growth retardants. Paclobutrazol treated plants have a reduced internodal length to give stouter stems and an enhanced root growth which provides anchorage. Paclobutrazol has been used to reduce shoot growth in rice by Syahputra et al. (2013) who found that an application of 400 mg/L at panicle initiation was optimum in reducing plant height and culm height, thus increasing bending resistance.

Thus, far, no attempt has been made to study the combined effect of the quasi-essential element, Si and gibberellins inhibitor, PBZ. Therefore, this study aims to explore the potential of silicon in enhancing the mechanical strength of the rice plant by looking at some agronomical traits, lignin content and elemental analysis besides deciphering its association with PBZ and verify if the combination of factors will outperform or remain the same in terms of yield components, growth and lodging resistance. Our argument is that a short plant with an increased rigidity will help our cause in overcoming the problem of lodging. However, the matter of importance is it should not affect the yield performance of the plant.

## Materials and methods

### Treatment and experimental design

The experiment was conducted in a randomized complete block design (RCBD) with three replicates to manage the error term and overcome the effect of heterogeneity of the field. The experiment was conducted to evaluate effect of Si and PBZ on yield and growth components with six treatments which are as follows:

Control (C)
Si applied at 4 g (4)
Si applied at 6 g (6)
Si applied at 4 g in combination of 400 mg/l PBZ (4T)
Si applied at 6 g in combination of 400 mg/l of PBZ (6T)
PBZ applied at 400 mg/l only (PBZ)

Silicon (SiO_2_ = 66%) in the form of fine powder and PBZ was applied at 57 days after sowing (DAS) as topdressing on soil surface and foliar spray, respectively. Amount applied is per pot.

### Crop establishment

The rice plants were grown in polyethylene containers with a diameter of 31.5 cm filled with ~15 kg of puddled soil that were obtained from Tanjung Karang, Selangor. The soil type is silty clay according to the classification of USDA Soil Taxonomy System (clay 52.11%, silt 46.76%, sand 1.08%). The pH, cation exchange capacity and Si content of the soil were 5.1, 15.63 meq/100 g soil, and 11.26 mg/kg, respectively. The seeds of MR219, a Malaysian rice variety were soaked in distilled water for 48 h, drained and allowed to germinate in petri dishes layered with wet filter paper for 24 h. Seeds with emerging radicles were then directly sown on moist soil surface with no standing water. A total of four seeds were sown in each container. This experiment was carried out in a glasshouse at Universiti Putra Malaysia, Selangor, Malaysia located at an altitude of 30 m above sea level, altitude of 30°21 N and longitude of 101°70 E. The humidity, minimum and maximum temperatures were 89.5%, 22 and 33.5°C, respectively.

### Growth parameters

Plant height was measured as the longest distance between the plant base and the tip of the highest leaf was measured 2 weeks before harvest. Internode length, panicle length, and culm length were measured too. Culm length was measured as the total length of all internodes or the distance between the plant base and the panicle necknode. Measurement was taken from eight plants per replicate for all parameters.

### Flag leaf area and chlorophyll content

Flag leaf area was measured using an Area Measurement System (Nebraska, USA). The sample was taken 2 weeks before harvest. Mean of leaf area was taken from five plants per replicate. Chlorophyll content of the flag leaf samples were measured using UV-Vis Scanning Spectrophotometer (Shimadzu). Samples with a leaf area of 3 cm^2^ were taken at 2 weeks before harvest and soaked in 20 ml of 80% acetone in the dark for 10 days to ensure the release of total chlorophyll from the tissue. Then, 3.5 ml of supernatant was sampled to measure the absorbance using a Spectrophotometer at 664 and 647 nm for the content of chlorophyll *a* and *b*, respectively. Mean of chlorophyll content was taken from five plants per replicate. The content of chlorophyll *a* and *b* were calculated according to the following formulas adopted from Coombs et al. (1985):

Chlorophyll *a* (mg/cm^2^) = (3.5/3) × (13.19 A_664_ – 2.57 A_647_)
Chlorophyll *b* (mg/cm^2^) = (3.5/3) × (22.10 A_647_ – 5.26 A_664_)
Total chlorophyll (mg/cm^2^) = Chlorophyll *a* + Chlorophyll *b*

### Yield and yield components

Yield components such as number of tillers, spikelets per panicle, percentage of filled spikelets, number of effective tillers, weight of 100 grains, and weight per panicle were measured. Sampling was done at harvest with eight plants per replicate.

### Quantification of silicon

#### Oxidization technique

Tissue analyses of Si were done according to autoclaved induced digestion (AID) method (Elliot and Synder, 1991). Each treatment was assessed in triplicates. Dried leaf and stem sample weighing 100 mg was transferred into an autoclave resistant polyethylene bottles which were previously rinsed with 0.1 M sodium hydroxide (NaOH) and deionized water. Next, the samples were wetted with 2 ml of 50% hydrogen peroxide (H_2_O_2_) followed by the addition of 5 ml of 50% (w/w) NaOH solution. Samples were loosely capped and autoclaved at 126 kPa for an hour. After autoclaving, samples were left to cool before adding 1 ml 5 mM of ammonium fluoride. Sample was diluted with distilled water to bring up the volume to 50 ml.

#### Colorimetric determination

Silicon content was determined colorimetrically. One milliliter of the sample solution was transferred into a 50 ml polyethylene bottle. This was followed by addition of 30 ml 20% acetic acid and 10 ml of ammonium molybdate solution (54 g/l, pH 7.0). After 5 min 5 ml of 20% tartaric acid and 1 ml of reducing agent was added and the volume was brought to 50 ml with 20% acetic acid. Reducing agent was prepared with 25 g of sodium bisulfite (NaHSO_3_) dissolved in 200 ml distilled water and this solution was added to another solution containing 2 g of sodium sulfite (Na_2_SO_3_), 0.4 g of 1-amino-2-naphtol-4-sulfonic acid in 25 ml of distilled water. The final volume was brought to 250 ml with distilled water (Elliott and Snyder, 1991). After 60 min the absorbance was measured with a spectrophotometer (UV-Visible; Varian, Australia) calibrated at 650 nm.

### Macronutrient analyses

Aboveground plant and leaf samples (third uppermost leaf) of the rice plant were dried in an oven at 65–75°C for 5 days. Samples were then ground to 1.00 mm sieve size followed by plant tissue digestion using HachDigesdahl Digestion which utilizes thermal convection wet digestion method (Hach et al., 1985). Briefly, 0.25 g of tissue sample was mixed into digestion tube with 5 ml of concentrated sulfuric acid (H_2_SO_4_). It was allowed to stand overnight. Next, 2 ml of 50% H_2_O_2_ was added down the sides of the digestion tube while rotating it. The digestion tubes were placed in the Digesdahl apparatus once the reaction had subsided at 285°C for 45 min. Samples were let to cool before adding 2 ml of H_2_O_2_ and then heated for 45 min at 285°C. This step was repeated once more or until the digests turn colorless or clear. The samples were then diluted with distilled water and made up to 100 ml in volume for analysis. Three replicates were used for each treatment. Nitrogen and phosphorus content were determined using QuickChem 8000 FIA Auto Analyzer (AA), whereas the contents of potassium, calcium and magnesium were determined with the use of Atomic Absorption Spectrophotometer (AAS).

### Lodging resistance

Lodging resistance was assessed by using a modified model of Kaack and Schwarz (2001). All data were recorded at harvest. Rice stems were cut at 25 cm from base of the plant and placed on a platform. The apparatus (Instron Ltd., Texture Analyzer) was equipped with a 5 kg load cell and a knife set with blade serving as a plunger to force the stem to bend with a velocity of 5.0 mm/s to a 45° angle. Three parameters which correspond to lodging resistance characteristics namely, hardness, stiffness, and brittleness were measured. Hardness indicates the force required to bend the sample to 45° whereas stiffness is the gradient of slope during bending while brittleness refers to the distance at which bending occurs.

### Lignin quantification

Thioglycolic acid lignin was determined according to modified method of Brinkmann et al. (2002). Dry ground leaf and stem plant sample was first washed with distilled water followed by 80% methanol extraction to isolate structural biomass for lignin analysis. Aliquots of 2 mg of structural biomass pellet were weighed into microcentrifuge tubes and mixed with 1.5 ml of 2 N hydrochloric acid (HCl) and 0.3 ml thioglycolic acid. Samples were then incubated at 95°C for 4 h and repeatedly mixed. Samples were rapidly cooled on ice and centrifuged for 10 min at 16,000 g. The supernatant was discarded. Pellets were washed with 1 ml distilled water. Thereafter, pellets were incubated with 1 ml of 0.5 N NaOH for 18 h on a shaker at room temperature. The suspension was centrifuged for 10 min at 16,000 g. The supernatant was carefully transferred into a new microcentrifuge tube. The pellet was re-suspended in 0.5 ml 0.5 N NaOH, vigorously mixed and centrifuged. The resulting supernatant was combined with the first alkaline supernatant and mixed with 0.3 ml concentrated HCl. Samples were incubated for 4 h at 4°C to precipitate the lignothioglycolate derivates. The samples were centrifuged, the supernatant discarded, and the pellet solubilized in 1 ml of 0.5 N NaOH. Absorbance of the resulting solution was measured at 280 nm using Multiskan Microplate Spectrophotometer Thermo Scientific USA. Calibration curves were generated by subjecting increasing amounts of 0.5–2.5 mg of commercial lignin (alkaline spruce lignin, Aldrich) to the same procedure.

### Scanning electron microscopy

Rice flag leaf samples taken 2 weeks before harvest were freeze dried prior to processing. Thereafter leaf segments were mounted on aluminum stubs previously covered with double-sided adhesive carbon tape and critical point dried. Next, samples were coated with gold in a sputter coater (Baic Tec Scd 005). Finally scanning electron microscope (LEO 1455 VPSEM Attached with EDX) at an accelerating voltage of 15 keV and magnification of 500 was used for morphological observation of Si associated structures in leaf epidermis. The percentage of silicon deposition was analyzed with an energy dispersive X-ray (EDAX) spectrometer combined with the microscope. Mapping of Si distribution was carried out on four treatments only (control, Si 4 g/pot, PBZ 400 ppm, Si 4 g+400 ppm PBZ) as cost saving measure and technical reason.

### Histochemical staining

Fresh leaf taken 2 weeks before harvest were fixed in formaldehyde acetic acid (FAA) and kept till viewing. The FAA was prepared by mixing 100 ml of 95% ethanol into 70 ml of distilled water before the addition of 20 ml of 37% formaldehyde and 10 ml of acetic acid. Phloroglucinol staining (flag leaf) was done on hand sectioned samples. The solution was prepared by dissolving 1.0 g of phloroglucinol in 40 ml of 20% ethanol before adding 20 ml of concentrated hydrochloric acid.

### Gene expression

Only four treatments, namely, control, plants treated with 4 g of Si/pot, PBZ 400 ppm/pot and 4T (4 g Si and 400 ppm PBZ) were analyzed as a cost saving measure and the two treatments that were omitted, 6 g of Si and 6T were rightfully represented by 4 g of Si and 4T, respectively. Total RNA of culm tissue taken 2 weeks before harvest in triplicates was extracted according to manufacturer's instruction (Total RNA Mini Kit Plant GeneAid). The RNA which has its maximum absorption peak at 260 nm was quantified *via* a Nanodrop. The ratio of the absorbance at 260/280 nm was used to assess the RNA purity of an RNA preparation. Total RNA samples were treated to remove genomic DNA. To an RNase-free microcentrifuge tube, 1 μg RNA was mixed with 1 μl of 10X Reaction Buffer with MgCl_2_, 1 μl of RNase-free DNase I and nuclease-free water made up to a volume of 10 μl. Samples were then incubated at 37°C for 30 min. Thereafter, 1 μl of 50 mM EDTA was added to terminate the reaction and re-incubated at 65°C for 10 min. These treated RNA samples were used in reverse transcription until which it was stored at −80°C.

### Reverse transcription

First strand cDNA was generated according to manufacturer's protocol (Thermo Scientific RevertAid First Strand cDNA Synthesis Kit). Real Time PCR was carried out according to manufacturer's protocol (Thermo Scientific Maxima SYBR Green qPCR Master Mix 2X). Briefly, 12.5 μl of SYBR Master Mix was added to 0.3 μM of forward and reverse primers before adding 100 ng of cDNA. Volume was made up to 25 μl with nuclease free water. Samples were loaded onto Bio-Rad CFX 96 with the following conditions: Initial denaturation at 95°C for 10 min followed by 40 cycles of denaturation at the same temperature for 15 s, annealing at 61°C for 30 s, and extension at 72°C for 30 s. Comparative method of C_T_ was used to calculate relative expression of gene (2^−ΔΔCT^; Schmittgen and Livak, 2008). Ubiquitin was used as the reference gene. Table 1 shows primer details.

**Table 1:** Detailed information of genes and primers used in this study.

**Genes**	**Primer sequence (5′–3′)**		**Product size (bp)**	**Accession number**
Cinnamyl-alcohol dehydrogenase (CAD)	Forward	AGTCGGTACTGTGTTGAG	158	Os02g0187800
	Reverse	GACGCTATGCAACAATCC		
Ubiquitin	Forward	GATCTTCGTGAAGACCCT	190	Os01g0328400
	Reverse	CGACTCCTTCTGGATGTT		

### Statistical analyses

All data were analyzed using the ANOVA procedure in the SAS Statistical software package Version 9.2. The differences among treatments were determined using the least significant difference (LSD) test at 0.05 probability level.

## Results

### Plant growth assessment

Plants treated with Si only were significantly taller than those treated with a combination of Si and PBZ. Untreated was not significantly different from Si treated plants but was found to be higher than all PBZ-treated plants (Table 2). All PBZ treated plants (4T, 6T, and PBZ) were statistically shorter in terms of culm length (Table 2). While the application of 4 g of Si was not significantly different from untreated, the culm length of the latter was also not statistically different from plants applied with 6 g of Si. Height and culm length of Si only treated plants were about 10 and 30% higher than PBZ treated plants. As for internode length, the results were comparable and showed similar trend as of height and culm length. Generally, untreated was not significantly different from plants treated with 4 and 6 g of Si (Table 3). However, all three treatments were significantly different from PBZ treated plants. Si only treated plants consistently showed higher values as compared to 4T, 6T, and PBZ. As indicated by Table 3, panicle length was not significantly different among treatments and measured in the range of 24–26 cm.

**Table 2:** Effect of silicon and paclobutrazol on height and culm length of rice MR219.

**Treatment**	**Height (cm)**	**Culm length (cm)**
C	115.9 ± 1.9^a^	75.1 ± 3.5^ab^
4	117.1 ± 5.1^a^	76.3 ± 2.8^a^
6	118.4 ± 3.6^a^	73.4 ± 0.4^b^
PBZ	106.1 ± 2.5^b^	56.4 ± 2.2^c^
4T	108.0 ± 2.6^b^	57.8 ± 1.4^c^
6T	105.7 ± 1.0^b^	57.6 ± 2.3^c^
LSD	5.28	2.44

*Means with same letter are not significantly different at p ≤ 0.05 according to Fisher's least significant difference (LSD). C, control; 4, 4 g Si/pot; 6, 6 g Si/pot; PBZ, PBZ only; 4T, 4 g Si+ 400 ppm PBZ; 6T, 6 g Si+ 400 ppm PBZ*.

**Table 3:** Effect of silicon and paclobutrazol on internode length and panicle length of rice MR219.

**Treatment**	**Internode length (cm)**					**Panicle length (cm)**
	**1st**	**2nd**	**3rd**	**4th**	**5th**	
C	30.6 ± 2.5^ab^	15.8 ± 0.2^a^	12.5 ± 0.2^a^	9.9 ± 0.5^a^	6.5 ± 0.5^b^	25.4 ± 0.4
4	31.4 ± 1.2^a^	16.3 ± 0.3^a^	11.8 ± 0.7^ab^	9.8 ± 0.7^a^	7.0 ± 0.5^a^	26.1 ± 0.6
6	29.2 ± 0.6^b^	16.0 ± 0.1^a^	12.4 ± 0.9^a^	9.9 ± 0.4^a^	6.0 ± 0.3^b^	25.8 ± 0.6
4T	23.6 ± 0.2^c^	11.8 ± 0.5^b^	10.8 ± 0.2^b^	7.1 ± 0.5^b^	4.5 ± 0.1^c^	25.4 ± 0.5
6T	23.7 ± 1.3^c^	12.1 ± 0.6^b^	10.8 ± 0.2^b^	6.8 ± 0.3^b^	4.3 ± 0.1^c^	24.9 ± 1.2
PBZ	22.5 ± 1.3^c^	11.9 ± 0.4^b^	11.0 ± 0.1^b^	6.6 ± 0.5^b^	4.4 ± 0.2^c^	24.5 ± 0.8
LSD	5.29	0.57	0.97	0.73	0.52	ns

*Means with same letter in the same column are not significantly different at the p ≤ 0.05 according to Fisher's least significant difference (LSD). ns, not significant; C, control; 4, 4 g Si/pot; 6, 6 g Si/pot; PBZ, PBZ only; 4T, 4 g Si+ 400 ppm PBZ; 6T, 6 g Si+ 400 ppm PBZ*.

### Flag leaf area and chlorophyll content

**Figure 1:**
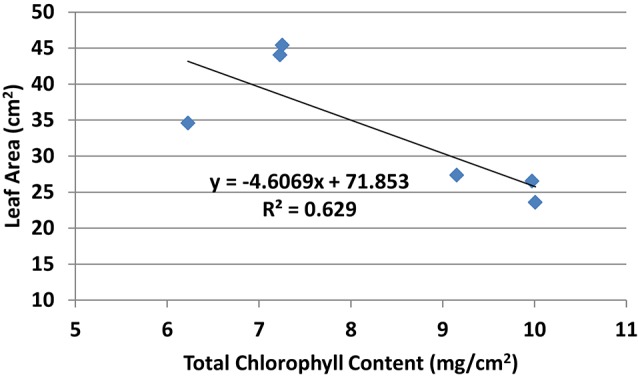
Relationship between total chlorophyll content and flag leaf area of rice MR219.

Chlorophyll *a*, chlorophyll *b*, and total chlorophyll content showed similar pattern whereby the chlorophyll content in general was higher in all PBZ treated plants than Si only treated plants (Table 4). All PBZ treated plants had highest chlorophyll content whereas control had the lowest content, but it was not significantly different from Si only treated plants. As for flag leaf area, Si-treated plants had a significantly larger surface area as compared to other treatments (Table 4). Leaf areas of plants treated with 4 g of Si and 6 g of Si were not significantly different. Application of PBZ only had the smallest leaf area at 23.58 cm^2^ though not significantly different from 4T and 6T. Application of Si only, resulted in an increase of about 27% in leaf area whereas it was reduced by 32% in plants applied with PBZ compared to control. However, flag leaf area of 4T and 6T was only reduced by 22%. Total chlorophyll content and flag leaf area were inversely proportional, the former increased when the latter was smaller (Figure 1).

**Table 4:** Effect of silicon and paclobutrazol on chlorophyll content and flag leaf area of rice MR219.

**Treatment**	**Chlorophyll content (mg/cm**^**2**^**)**			**Flag leaf area (cm^2^)**
	***a***	***b***	**Total**	
C	4.63 ± 0.75^b^	1.59 ± 0.25^b^	6.23 ± 1.00^b^	34.58 ± 0.98^b^
4	5.35 ± 0.24^b^	1.88 ± 0.14^b^	7.23 ± 0.38^b^	44.04 ± 2.80^a^
6	5.42 ± 0.64^b^	1.83 ± 0.26^b^	7.25 ± 0.90^b^	45.39 ± 2.41^a^
PBZ	7.45 ± 0.93^a^	2.56 ± 0.45^a^	10.01 ± 1.38^a^	23.58 ± 3.85^c^
4T	6.77 ± 1.81^a^	2.38 ± 0.65^a^	9.16 ± 2.44^a^	27.35 ± 4.59^bc^
6T	7.37 ± 1.33^a^	2.60 ± 0.45^a^	9.98 ± 1.78^a^	26.52 ± 3.38^bc^
LSD	1.141	0.410	1.526	9.40

*Means with same letter in the same column are not significantly different at p ≤ 0.05 according to Fisher's least significant difference (LSD). C, control; 4, 4 g Si/pot; 6, 6 g Si/pot; PBZ, PBZ only; 4T, 4 g Si+ 400 ppm PBZ; 6T, 6 g Si+ 400 ppm PBZ*.

### Yield and yield components

**Figure 2:**
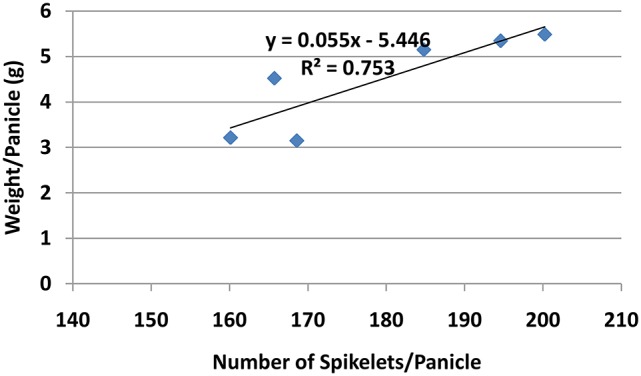
Relationship between spikelets per panicle and weight per panicle of rice MR219.

Though the percentage of effective tillers of plants treated with Si only (4 and 6 g/pot) were not statistically different from untreated, they were significantly higher than all treatments corresponding to PBZ treated plants (Table 5). While the application of 4 and 6 g of Si showed 3% increase in percentage of effective tillers, it was the opposite in PBZ only treated plants whereby 4% reduction was observed, in comparison to untreated. In 4T and 6T it was reduced by 10%. Statistical analysis revealed application of Si at 4, 6 g, and PBZ resulted in a panicle weight in the range of 5 g per panicle and were significantly different as compared to other treatments (Table 5). 4T and 6T had the lowest weight and were not significantly different from each other. As for number of spikelets per panicle, plants treated with Si only (4 and 6 g) showed highest number of grains followed by those treated with PBZ only, though the latter was not significantly from plants treated with 4 g of Si (Table 5). The number of spikelets per panicles showed 19% and 11% increase in plants treated with Si and PBZ only, respectively. There was a significant positive correlation between number of spikelets per panicle and weight per panicle with a *r*-value of 0.87 (Figure 2). On the other hand, percentage of filled spikelets and weight of 100 grains were not significantly different in plants treated with 6 g of Si, 4 g of Si, and PBZ alone. In terms of the former trait, it showed a mere increase of 2% in plants treated with Si and PBZ as single factor but reduced by 7% in 4T and 6T compared to control. Application of either Si or PBZ alone resulted in highest percentage of filled spikelets (Table 5). Untreated plants had a higher percentage of filled spikelets than plants treated with a combination of Si and PBZ (4T and 6T). As for weight of 100 grains, 4T and 6T weighed the lowest (Table 5). Plants treated with 4 g of Si, 6 g of Si, PBZ and control showed comparable means as they were not significantly different.

**Table 5:** Effect of silicon and paclobutrazol on yield components of rice MR219.

**Treatment**	**Effective tillers (%)**	**Weight/Panicle (g)**	**No of spikelets/Panicle**	**Filled spikelets (%)**	**Weight 100 grains (g)**	**Number of tillers**
C	95.93 ± 1.42^ab^	4.52 ± 0.13^b^	166 ± 5.8^c^	85.75 ± 1.99^b^	2.85 ± 0.06^a^	41 ± 1^c^
4	99.27 ± 1.26^a^	5.34 ± 0.22^a^	195 ± 1.0^ab^	87.90 ± 2.02^a^	2.98 ± 0.06^a^	47 ± 2^b^
6	98.07 ± 1.92^a^	5.49 ± 0.27^a^	200 ± 3.4^a^	87.67 ± 1.37^a^	2.99 ± 0.10^a^	50 ± 2^a^
PBZ	92.03 ± 1.12^bc^	5.15 ± 0.40^a^	185 ± 5.5^b^	87.73 ± 0.81^a^	2.88 ± 0.17^a^	46 ± 1^b^
4T	86.83 ± 5.69^cd^	3.22 ± 0.42^c^	160 ± 9.4^c^	78.46 ± 2.42^d^	2.09 ± 0.17^b^	43 ± 2^c^
6T	85.87 ± 3.79^d^	3.15 ± 0.44^c^	169 ± 3.6^c^	81.27 ± 1.19^c^	1.95 ± 0.22^b^	42 ± 1^c^
LSD	5.30	0.541	10	1.88	0.273	2.52

*Means with same letter in the same column are not significantly different at p ≤ 0.05 according to Fisher's least significant difference (LSD). C, control; 4, 4 g Si/pot; 6, 6 g Si/pot; PBZ, PBZ only; 4T, 4 g Si+ 400 ppm PBZ; 6T, 6 g Si+ 400 ppm PBZ*.

### Silicon content

Silicon content was significantly different between treatments in both leaf and aboveground plant samples. Plants treated with 6 g of Si had a significantly higher Si content as compared to plant treated with 4 g of Si in leaf samples (Table 6). These treatments were followed by 4T and 6T. Untreated and PBZ only treated plants had significantly lowest Si content. As for aboveground plant samples, Si content of plants treated with 6 and 4 g of Si were significantly higher than all other treatment combinations (Table 6). Untreated and PBZ only treated plants had lowest value as in the case of leaf samples.

**Table 6:** Effect of silicon and paclobutrazol on silicon and lignin content in leaf and aboveground plant samples of rice MR219.

**Treatment**	**Silicon content (g/kg)**		**Lignin content (mg/g)**	
	**Leaf**	**Stem**	**Leaf**	**Stem**
C	14.06 ± 0.08^d^	14.43 ± 0.08^b^	117.87 ± 5.95^c^	99.50 ± 9.12^d^
4	14.62 ± 0.04^b^	15.37 ± 0.11^a^	155.74 ± 1.64^a^	140.32 ± 6.41^a^
6	14.91 ± 0.11^a^	15.40 ± 0.08^a^	147.45 ± 8.60^ab^	129.77 ± 5.93^ab^
PBZ	14.05 ± 0.10^d^	13.72 ± 0.20^c^	132.15 ± 4.91^bc^	100.65 ± 9.17^cd^
4T	14.38 ± 0.10^c^	14.54 ± 0.39^b^	139.68 ± 5.02^ab^	120.46 ± 5.78^abc^
6T	14.44 ± 0.11^c^	14.49 ± 0.13^b^	133.25 ± 4.64^bc^	112.27 ± 2.31^bcd^
LSD	0.157	0.681	17.389	20.084
**SAMPLE TYPE**				
Aboveground	14.66 ± 0.68^a^		117.16 ± 18.09^b^	
Leaf	14.41 ± 0.32^b^		137.69 ± 14.42^a^	
Pr> F	^*^		^***^	

Means with same letter in the same column are not significantly different at p ≤ 0.05 according to Fisher's least significant difference (LSD). C, control; 4, 4 g Si/pot; 6, 6 g Si/pot; PBZ, PBZ only; 4T, 4 g Si+ 400 ppm PBZ; 6T, 6 g Si+ 400 ppm PBZ.

* *P ≤ 0.05*.

*** *P ≤ 0.001*.

### Lignin content

The lignin content of both leaf and aboveground samples were significantly different (Table 6). For the former, plants treated with 4 g of Si showed the highest value though not significantly different from plants treated with 6 g of Si and 4T. As for PBZ and 6T, they were not significantly different from untreated. Likewise, lignin content of 4 g of Si, 6 g of Si, and 4T of aboveground samples were significantly not different and were significantly higher than other treatments. Lignin content of plants treated with 6 g of Si was not statistically different from 4T, 6T, and untreated. Factorial analyses showed lignin content of leaf samples were significantly higher than aboveground.

### Macronutrient analyses

Nitrogen content in plant tissues was not significantly different in terms of treatment in both leaf and aboveground samples. However, leaves had significantly higher nitrogen content as compared to aboveground. Content in leaves and aboveground samples ranged from 3.64 to 3.11% and 2.31 to 2.02%, respectively (Table 7). Phosphorus content was not significantly different in both types of samples (Table 7). In contrast to nitrogen content, aboveground was significantly higher than leaves. Potassium in leaves were not significantly different between treatments, however it was significantly different in aboveground samples (Table 7). Plants treated with PBZ only showed lowest accumulation which was significantly different than the rest of the treatments. Meanwhile, factorial analysis revealed aboveground had significantly higher potassium content than leaves. Calcium content was significantly different in leaf samples (Table 7). 4T had the highest content followed by 6T and PBZ; all three treatments were not statistically different. Untreated was not significantly different from plants treated with 4 g of Si, 6 g of Si, and PBZ only. However, calcium content was not significantly different in aboveground samples. Factorial analysis showed leaf samples as having significantly higher calcium content compared to aboveground samples. There was one-fold increase in the content in leaf samples. Magnesium content was not significantly different between treatments in both leaf and aboveground samples (Table 7). Factorial analysis proved magnesium content was significantly higher in leaf compared to aboveground samples.

**Table 7:** Effect of silicon and paclobutrazol on macronutrients in leaf and aboveground plant samples of rice MR219.

**Treatment**	**Content (%)**									
	**Leaf**					**Aboveground**				
	**N**	**P**	**K**	**Ca**	**Mg**	**N**	**P**	**K**	**Ca**	**Mg**
C	3.38 ± 0.43	0.27 ± 0.02	2.33 ± 0.87	0.26 ± 0.15^c^	0.18 ± 0.08	2.22 ± 0.53	0.33 ± 0.01	3.28 ± 0.25^a^	0.12 ± 0.02	0.33 ± 0.03
4	3.64 ± 0.71	0.28 ± 0.00	2.85 ± 0.39	0.24 ± 0.11^c^	0.20 ± 0.06	2.31 ± 0.43	0.31 ± 0.03	3.35 ± 0.36^a^	0.13 ± 0.01	0.31 ± 0.02
6	3.47 ± 0.95	0.27 ± 0.03	3.05 ± 0.63	0.27 ± 0.14^bc^	0.19 ± 0.08	2.22 ± 0.58	0.31 ± 0.06	3.34 ± 0.59^a^	0.12 ± 0.03	0.31 ± 0.03
4T	3.31 ± 0.35	0.25 ± 0.01	2.81 ± 0.47	0.34 ± 0.19^a^	0.21 ± 0.07	2.03 ± 0.05	0.29 ± 0.02	3.07 ± 0.37^a^	0.15 ± 0.04	0.29 ± 0.01
6T	3.11 ± 0.63	0.26 ± 0.03	2.76 ± 0.22	0.33 ± 0.16^ab^	0.20 ± 0.07	2.02 ± 0.43	0.30 ± 0.03	3.02 ± 0.37^a^	0.16 ± 0.01	0.30 ± 0.01
PBZ	3.48 ± 0.64	0.29 ± 0.02	2.73 ± 0.67	0.28 ± 0.14^abc^	0.22 ± 0.07	2.14 ± 0.02	0.35 ± 0.02	2.60 ± 0.29^b^	0.13 ± 0.02	0.35 ± 0.02
LSD	ns	ns	ns	0.064	ns	ns	ns	0.408	ns	ns
	**N**		**P**		**K**		**Ca**		**Mg**	
Aboveground	2.16 ± 0.36^b^		0.31 ± 0.03^a^		3.11 ± 0.42^a^		0.13 ± 0.03^b^		0.15 ± 0.02^b^	
Leaf	3.4 ± 0.57^a^		0.27 ± 0.02^b^		2.75 ± 0.54^b^		0.29 ± 0.13^a^		0.20 ± 0.06^a^	
Pr > F	^***^		^***^		^***^		^***^		^**^	

Means with same letter in the same column are not significantly different at p ≤ 0.05 according to Fisher's least significant difference (LSD). C, control; 4, 4 g Si/pot; 6, 6 g Si/pot; PBZ, PBZ only; 4T, 4 g Si+ 400 ppm PBZ; 6T, 6 g Si+ 400 ppm PBZ.

** *P ≤ 0.01*,

*** *P ≤ 0.001. ns, not significant*.

### Bending resistance

Hardness of plants treated with Si or PBZ alone were not significantly different and were in the order of 4 g of Si, PBZ, and 6 g of Si (Table 8). 4T and 6T did not outperform control plants. As for stiffness, no significant differences observed among treatment though values followed the pattern of hardness. As for brittleness, PBZ showed highest value followed closely by plants treated with 4 and 6 g of Si; though all three were not significantly different from one another. There was a significant positive relationship between hardness and stiffness as indicated by its coefficient of 0.89.

**Table 8:** Bending resistance of rice MR219 in relation to application of silicon and paclobutrazol.

**Treatment**	**Hardness (g)**	**Stiffness (g/mm)**	**Brittleness (mm)**
C	1207.5 ± 13.5^bc^	249.9 ± 30.4	4.651 ± 0.1^c^
4	1548.9 ± 20.4^a^	333.9 ± 10.4	4.966 ± 0.4^ab^
6	1313.4 ± 16.3^ab^	260.2 ± 33.4	4.859 ± 0.1^ab^
PBZ	1355.0 ± 17.2^ab^	259.7 ± 23.1	5.065 ± 0.3^a^
4T	986.8 ± 22.3^c^	206.2 ± 44.9	4.191 ± 0.2^c^
6T	1156.4 ± 18.5^bc^	245.7 ± 27.7	4.631 ± 0.1^b^
L.S.D.	287.19	ns	0.3535

*Means with same letter in the same column are not significantly different at p ≤ 0.05 according to Fisher's least significant difference (LSD). ns, not significant. C, control; 4, 4 g Si/pot; 6, 6 g Si/pot; PBZ, PBZ only; 4T, 4 g Si+ 400 ppm PBZ; 6T, 6 g Si+ 400 ppm PBZ*.

### Real time PCR

**Figure 3:**
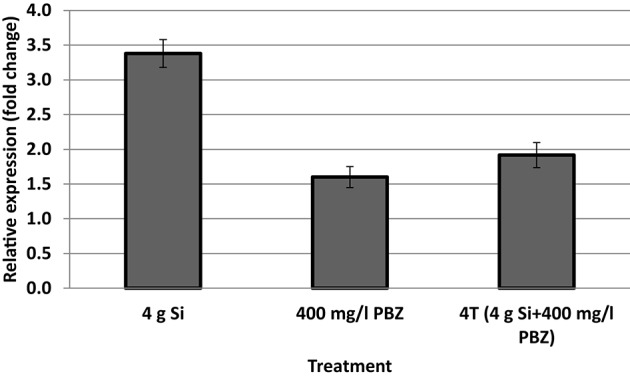
mRNA transcript abundance of cinnamyl alcohol dehydrogenase.

Only samples with acceptable OD values and RNA concentration of above 100 ng/μl were used for cDNA synthesis. Expression of CAD was up-regulated 3.4-fold in plants treated with 4 g of Si (Figure 3) whereas the fold change was lowest in PBZ at 1.6. This was even lower than 4T which was up-regulated 1.9-fold.

### SEM observations

**Figure 4:**
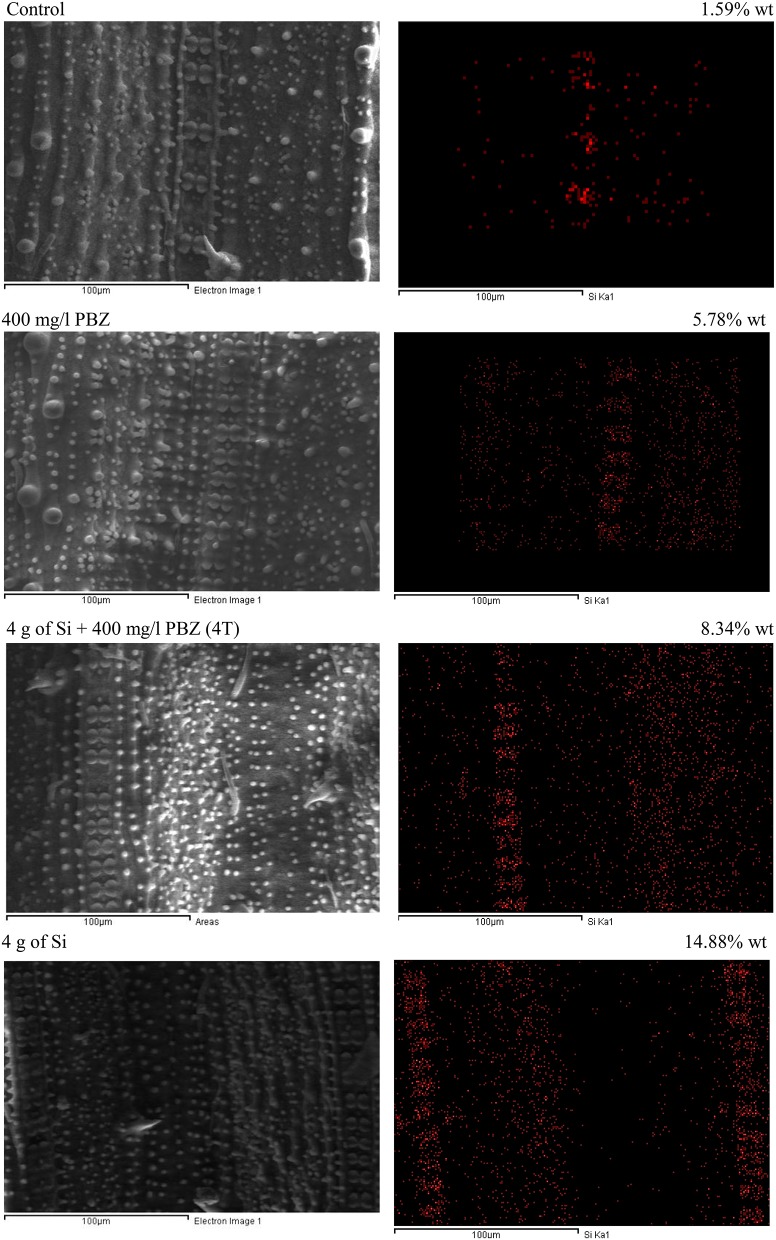
Scanning electron micrographs of silicon mapping and distribution of rice MR219 leaves. **Right column:** scanning electron micrograph. **Left column:** corresponding silicon map.

Si mapping via SEM showed the distribution of Si on leaf samples in four treatments namely, control, plants treated with 4 g of Si, PBZ, and plants treated in combination of 4 g of Si and PBZ (4T) (Figure 4). Si distribution in untreated sample was the least (1.59% wt) as compared to other treatments. It was only centered around the row of silica cells with minimal scattering of small silica bodies. PBZ treated sample showed a slight enhancement (5.78%) in Si abundance. Leaf sample of plants treated with both Si and PBZ showed a very high distribution of Si (8.34%) in a row of silica cells and small silica bodies all around the sample. Si only treated plants showed the highest Si distribution at 14.88% as compared to other treatments. It had two full rows of silica cells and Si was found throughout the sample.

**Figure 5:**
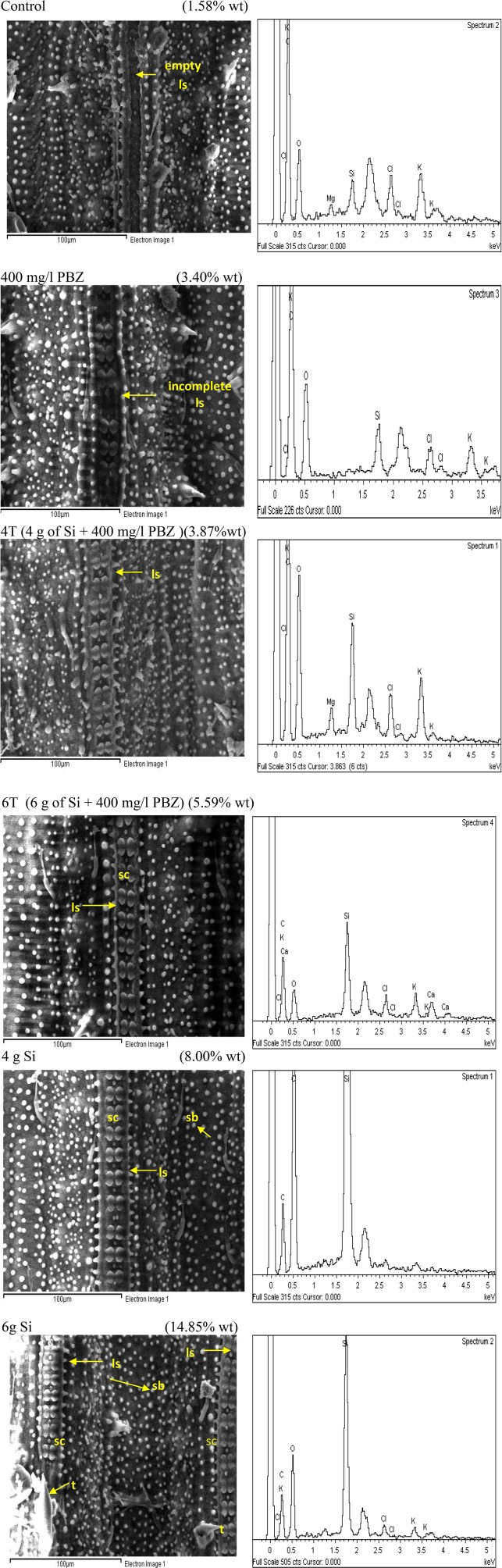
SEM and the respective EDX spectra of leaf adaxial surface of rice MR219. sc, silica cell; sb, silica bodies; ls, ladderlike structure; t, trichome.

Adaxial surface of untreated plants showed the lowest Si weight at 1.58% followed by PBZ treated plants (3.4%; Figure 5). The former clearly lacked dumb-bell shaped silica cells as the ladder-like structure was empty whereas the latter showed the presence of ladder-like structure with incomplete silica cells. Treatments 4T and 6T followed next with Si weight of 3.87 and 5.59%, respectively. Both showed a complete row of dumb-bell shaped silica cells with varying degree of small silica bodies. Plants treated with Si alone showed highest distribution of Si at 8 and 14.85% in plants treated with 4 and 6 g of Si, respectively. The latter possessed two ladder-like structures complete with silica cells, bulky scales, and pricky hair protuberance whereas the former only had a single row of dumb-bell shaped silica cells and pricky hair protuberance but lacked the bulky scale-like trichomes. EDX spectra analysis indicated heavy presence of Si on scale-like trichomes, pricky hair, silica cells, and small silica bodies in plants treated with 4 and 6 g as compared to the other four treatments.

**Figure 6:**
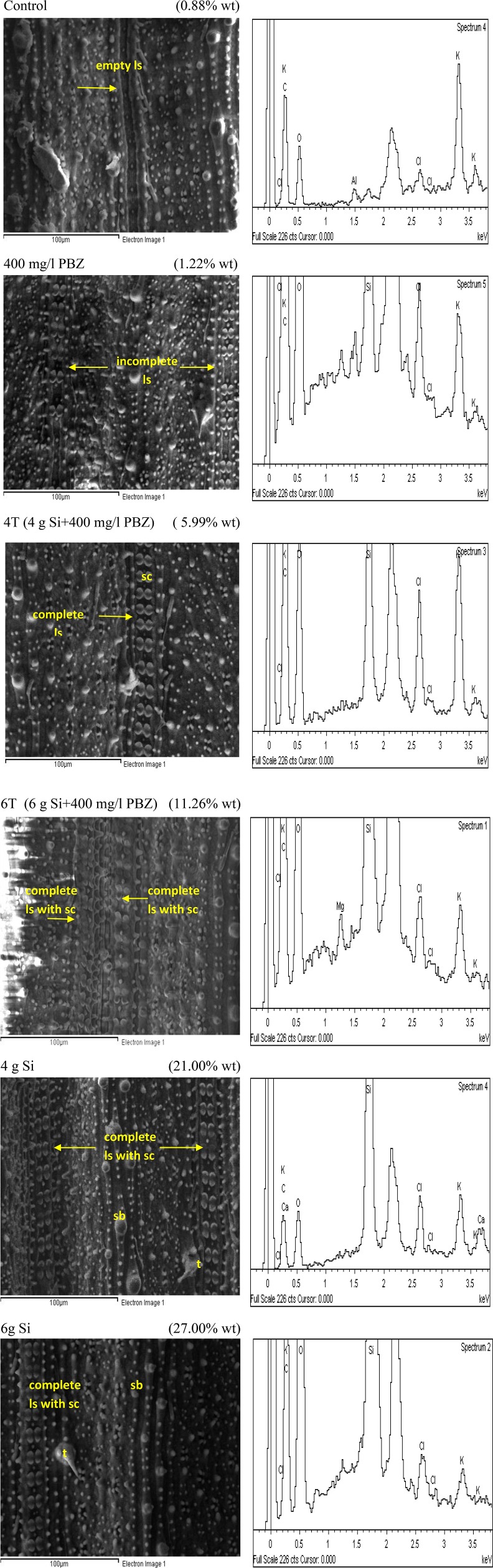
SEM and the respective EDX spectra of leaf abaxial surface of rice MR219. sc, silica cell; sb, silica bodies; ls, ladderlike structure; t, trichome.

Similar to adaxial, untreated plants showed the lowest Si deposition in abaxial surface at 0.88% (Figure 6). It completely lacked dumb-bell shaped silica cells while the many silica bodies observed did not show any significant Si deposition. This was followed by plants treated with PBZ only with a weight of 1.22% which showed presence of ladder-like structure with incomplete silica cells. Meanwhile, Si incorporation in 4T and 6T were enhanced with a value of 5.99 and 11.26%, respectively. The latter has double rows of dumb-bell shaped silica cells and more small silica bodies as compared to the other. However, plants treated with Si only (4 and 6 g Si) showed highest deposition of Si at 21 and 27%. Si was prominently present on trichomes and silica bodies.

### Anatomical examination

**Figure 7:**
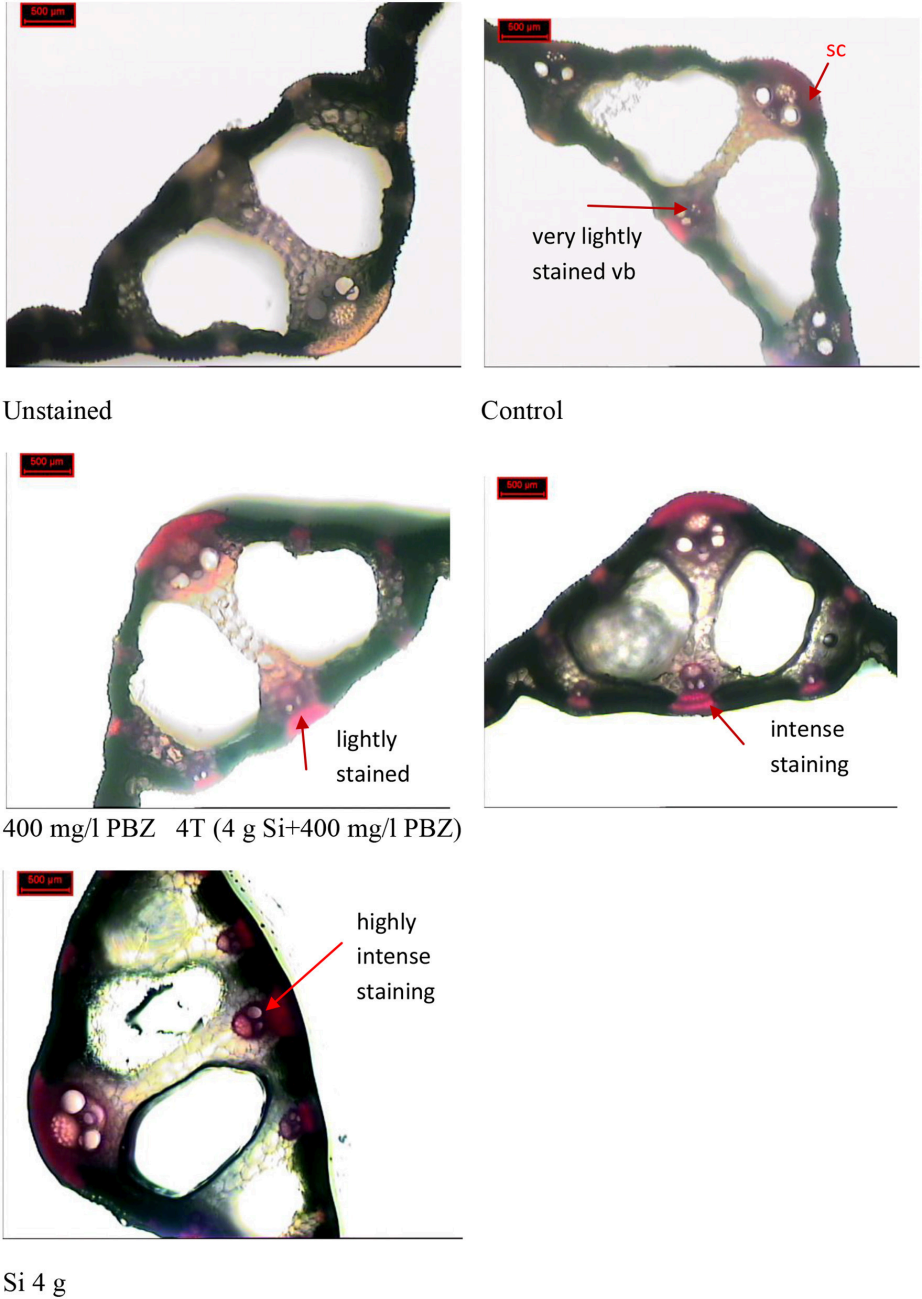
Phloroglucinol-HCl staining of leaf cross section. vb, vascular bundle.

Phloroglucinol staining which stains lignin red, showed presence of lignified vascular bundles in leaf cross section of all plants regardless of treatment (Figure 7). However, there were differences in terms of intensity and thickness. In the control plant, vascular bundles were stained very lightly and at some places were nearly invisible. This was in contrast to the other three treatments (PBZ, Si, and 4T) which showed clearly stained vascular bundles and sclerenchyma band. The latter was present in the form of fiber cells around the vascular bundles. In plants treated with Si only, vascular bundles and sclerenchyma band displayed the most intense staining.

## Discussion

Plant growth regulators are found to have many practical applications in controlling vegetative and reproductive growth and physiological activities of plant (Jaleel et al., 2007). PBZ which is a gibberellin synthesis inhibitor have long been used in agriculture to induce flowering, fruiting, ripening, and reducing lengthening. In the current study, all length related parameters such as height, culm length, and internode length were reduced in PBZ treated plants as reported by Yim et al. (1997) and Berova and Slate (2000). PBZ interfered with ent-kaurene oxidase activity in the ent-kaurene oxidation pathway (Rademacher, 1997) which led to inhibition of gibberellin biosynthesis and abscisic acid catabolism. PBZ is involved in internode shortening, induces shoot growth reduction (Terri and Millie, 2000; Sebastian et al., 2002), long term growth suppression in both monocot and dicot (Rahman et al., 1989) and reduction in vegetative growth and canopy area in mango (Blaikie et al., 2004) and apple (Wani and Lone, 2007).

Besides, reduced cell proliferation could be responsible for restricted shoot growth (Haughan et al., 1989). The results obtained in this study were in agreement to Tekalign and Hammes (2005) who noticed a reduction in shoot growth of PBZ treated potato which resulted in a short and compact plant. They hyphothesized that it could be attributed to reduction in total leaf area and stem elongation. This is true as PBZ treated plants showed higher chlorophyll content compared to untreated and Si-treated plants in this study. The total chlorophyll content which includes chlorophyll *a* and *b* nearly doubled in plants treated with PBZ only compared to untreated. Similar results were obtained in PBZ treated barley seedlings (Sunitha et al., 2004) and tomato (Still and Pill, 2003) whereby chlorophyll content was two-fold higher than untreated.

On the contrary, flag leaf area was highest in plants treated with 4 and 6 g of Si. In fact, leaf area and chlorophyll content was negatively correlated at −0.71. The leaf area of these treatments were much higher than PBZ treated plants due to the incorporation of Si in the treatment which is known to keep leaves erect, thus increasing surface area. It is postulated that leaves of PBZ treated plants might be thicker as they seem to possess high chlorophyll content though leaf areas are much smaller. Microscopy observation verified that thicker leaves of treated plants were due to the induction of elongated and thicker epidermal cells, thicker palisade and spongy mesophyll tissue (Tekalign and Hammes, 2005). On the other hand, Khalil and Rahman (1995) through their work on corn found that densely packed chloroplasts over small leaf area resulted in increased chlorophyll content. Besides, an increased leaf thickness in response to PBZ treatment was found in maize (Sopher et al., 1999), chrysanthemums (Burrows et al., 1992), and wheat (Gao et al., 1987).

In terms of yield components, effective tillers of PBZ treated plants showed a lower percentage than Si treated plants. Overall, plants treated with PBZ alone performed on par with those treated with Si only though not any better in terms of yield components. 4T and 6T did not show any significant yield or growth improvement. As mentioned earlier, PBZ counters vegetative growth and enhanced seed setting by changing assimilate partitioning.

In okra, PBZ is reported to be involved in accumulation of carbohydrates thus it hastens a rapid growth of sinks or pods where it significantly increased crop yields ha^−1^ (Whiley, 1993; Katz et al., 2003; Chutichudet et al., 2006). In rice, application of 50 mg L^−1^ PBZ at the heading stage increased number of spikelets per panicle, seed setting rate and grain yields in two local cultivars tested (Pan et al., 2013). Likewise, Peng et al. (2011), found that spraying PBZ in rice had more effective grain number, seed setting rate, 1,000-grain weight, and yield was increased by 11.89% under PBZ treatment compared to untreated. According to Sun (2004) PBZ did not affect gaseous exchange, thus photosynthetic rate was not affected. He then suggested that this growth regulator could be used in upland rice to reduce height and prevent lodging without affecting productivity. However, he did mention that this may not hold true in all cases and results may differ if higher rates are used. In contrast, Alvarez et al. (2012) reported a close relationship between yield components and grain yield; and between these and plant height. He mentioned that height reduction in plants also resulted in negative consequences for yield components, with direct consequence on grain yield which was also evident in this study. Li et al. (2017) concluded that direct seeded rice treated with paclobutrazol reduced yield and showed negative effect on weight of filled grains and other yield related parameters.

Meanwhile, the three bending parameters measured, hardness, stiffness, and brittleness showed that lodging resistance were higher in plants treated with either Si or PBZ. Combination 4T and 6T did give satisfactory results but weak plant structure. Fallah (2012) noticed increased bending moment, breaking resistance and lower lodging index in plants supplied with Si as opposed to control. Deposition of Si could enhance stem strength by forming Si-cuticle double layer beneath epidermal cells, increasing number of silicified cells and accumulating more Si in culm and thus resist lodging. In cereal crops such as rice and wheat, PBZ had been used to control lodging by reducing vegetative growth (Froggatt et al., 1982) and not on improving seed setting. According to Ookawa and Ishihara (1992), lodging resistance of lodging prone varieties had been improved by decreasing the length and weight of the above-ground parts of the plant. Nonetheless, application of plant growth regulators that reduces stem length may not necessarily reduce lodging but only delay onset of lodging as shown in barley and wheat (Ma and Smith, 1992).

Yield components and bending resistance of plants treated with a combination of Si and PBZ did not show improvement as compared to application of these treatments as single factor. Initially, it was thought the effects of PBZ and Si would be additive, however we observed a deviation from expected. Epistasis, which affects the phenotypic expression of genes due to interactions between non-allelic genes (Li et al., 1997), may have resulted in these results. If the effect of one locus is altered or camouflaged by effects at another locus, the power to detect the first locus is likely to be reduced and elucidation of the joint effects at the two loci will be hindered by their interaction (Cordells, 2002). Fisher (1930) termed epistasis as the statistical deviation from the additive combination of two loci in their effects on an expression of trait. According to Hao et al. (2012), most described effects using plant growth regulators are indirect which may be due to secondary effects as induced by the treatment at the site of application. Besides, yield components fall under the category of polygenic traits displaying continuous variation. Similarly, bending resistance is dependant upon the strength of elongated internodes which is affected by mechanical strength, chemical composition, and plant nutritional status. The mechanical strength on the other hand is related to culm thickness and tissue strength and is affected by growing conditions (Yoshida, 1981). Thus, epistatic interactions of genes involved in culm strength and yield should be further explored.

Lignin content of all Si-treated plants in both type of plant samples were higher than untreated. Silicon induces production of phenolic compounds such as lignin (Rodrigues et al., 2005). Besides, Inanaga et al. (1995), reported that Si has high affinity for organic polyhydroxyl compounds that are involved in the biosynthesis of lignin. In addition, Si is reported to play an important role in phenolic metabolism and the biosynthesis of lignin in cell walls (Marschner, 1995). Furthermore, results from qPCR clearly showed significant up-regulation of CAD gene in plants treated with Si alone as opposed to control. This result could be associated with lignin content as CAD is a gene of utmost importance in the finals steps of monolignol synthesis that catalyzes the reduction of cinnamyl aldehyde to cinnamyl alcohol prior to polymerization into the lignin polymer. Wang et al. (2014) reported an increase in lignin content and up-regulation of CAD gene in buckwheat which resulted in lodging resistance.

Mutant plants are often utilized for functional studies. For example, in *gh2* mutant of an unknown background showed lower *OsCAD2* gene expression and lignin content compared with a control rice plant (Ookawa et al., 2008). This clearly indicates that downregulation of genes encoding biosynthetic pathway often leads to a reduction in lignin content. In this study, plants treated with Si showed up-regulation of CAD accompanied by higher lignin content. Lignin biosynthesis genes are expected to be highly expressed in stems, where secondary cell walls are prevalent and lignification occurs, while remaining at relatively low levels in roots and especially leaves (Trabucco et al., 2013). A noteworthy point is that mutations in CAD could lead to pleiotropic effects of dwarfing, lodging, and a decrease in grain and biomass yield (Sattler et al., 2010). In the grass family of Poaceae, CAD genes are expressed in vascular bundles and are related to the mechanical properties of the stem tissues (Fan et al., 2009).

Histochemical analysis revealed intense lignin accumulation in Si treated plants in the regions encircling vascular bundle in addition to sclerenchyma layer beneath the epidermis. This further supports the results obtained from qPCR and thioglycolic acid lignin analysis. In rice leaves, Si is deposited in the epidermis, vascular bundles plus bundlesheath, and sclerenchyma (Kim et al., 2002). As such, it is postulated that Si may have induced lignin accumulation in this study as evident by histochemical staining for the complex polymer. Early studies showed that the lodging-resistant types of rice had a thicker band of sclerenchyma at the periphery of the stem compared with lodging-susceptible strains (Ramaiah and Mudaliar, 1934). Meanwhile recent studies showed that lodging-resistant rice varieties had more vascular bundles in both the peripheral and the inner section of the outer layers, as compared to lodging-susceptible varieties (Chaturvedi et al., 1995). Earlier experiments in this study found that application of Si improved lodging resistance (Dorairaj et al., 2017). These findings indicate that the thickness of cell walls in the sclerenchyma and the number of vascular bundles are important factors that affect the stem mechanical strength of rice.

Silicification takes place by means of phytoliths deposition. Silicified cells take shape in the form of silica cell and silica body which is also known as silica motor cell (Ma, 1990). Dumb-bell shaped silica cells are often located on vascular bundles whereas silica bodies are located in bulliform cells of rice leaves (Ma and Yamaji, 2006). These bulliform cells are involved during water stress as it aids the leaves to roll up under stressed condition. On the other hand, silica bodies in grasses are thought to function as support structure (Piperno and Pearsall, 1998). Trichomes which range from glandular to non-glandular and unicellular to multicellular, can take many forms in plants each suited to its function. These sharp appendages play roles in both chemical and physical defense against attacks from pests and insects thus increasing disease resistance.

Macroscopic examinations are tools of evidence and offers much input. In this study, SEM/EDX analysis showed plants treated with Si had the highest Si abundance and distribution. Si was mainly deposited in small silica bodies, dumb-bell shaped silica cells, and trichomes in Si-treated plants. Dumb-bell shaped silica cells were arranged on ladder-like structures. However, in control and plants treated with PBZ only, Si was very low and not deposited on these structures. This indicates that presence of Si associated structure does not guarantee Si deposition. Yamanaka et al. (2009) reported that ladder-like structure which is primarily involved in mechanical strengthening of epidermis, inhibited flat leaves from rolling and as such may aid in efficient light absorption for photosynthesis thus improving growth. Meanwhile, Wang et al. (2005) revealed that leaf temperature is very much reduced in presence of silicified cells via emission of infrared radiation, thus reducing water loss during transpiration. Si enriched trichomes together with silica cells and silica bodies impart strength and support for plant architecture and serves as mechanical barrier against probing and chewing by insects such as stem borer and planthopper (Ma and Yamaji, 2006).

Meanwhile, Saigusa et al. (2000) reported that Si content in rice leaf blades corrected with that of silicified bulliform cells and trichomes. Another interesting finding was that Si deposition moves from small cells to bulliform cells and trichomes as Si content increases. This is applicable to the current study as SEM/EDX analysis revealed that in plants with low Si concentration (untreated and PBZ), trichomes and silica bodies did not show significant Si weight. Ma and Yamaji (2006) reported that Si is involved in both physical strengthening and defense by forming silicified bulliform, long, and short cells in the leaf epidermis. These cells are formed beneath the cuticle to form a double layer which acts as a physical barrier. In addition, this layer is able to impede penetration by fungi and, thus disrupting the infection process (Ma and Yamaji, 2006). Besides, soluble silicon acts as a modulator of host resistance to pathogens by triggering a broad range of natural defenses (Currie and Perry, 2007). For example in Si treated cucumbers, the activity of pathogenesis related (PR) enzymes such as chitinases, peroxidases, polyphenol oxidases and flavonoid phytoalexins were enhanced and thus offered protection against fungal attacks (Fawe et al., 1998). Likewise in rice, differential accumulation of glucanase, peroxidase, and PR transcripts were associated with limited colonization by the fungus *Magnaporthe grisea* in epidermal cells of a susceptible rice cultivar treated with silicon (Rodrigues et al., 2005). Generally, defense mechanisms triggered by silicon include the accumulation of lignin, and generally, phenolic compounds, as well as chitinases and peroxidises (Ma and Yamaji, 2006).

This study was undertaken to test the effectiveness of Si when combined with a growth retardant such as PBZ. It was thought that this combination would result in a stronger plant structure that could reduce lodging and improve growth and yield components. Paclobutrazol gave comparable results to application of Si when used as single factor. This study could pave ways for other researches to study the combination of culm strengthening Si and other antagonists of gibberellin synthesis. It is possible to formulate the right combination to counter the loss of yield through lodging and biotic stresses. Though mapping epistatic interactions is challenging experimentally, statistically, and computationally, it should be undertaken to understand the mechanism underlying the outcome of this study.

## Conclusion

It is clear that silicification can strengthen the epidermal cell wall and improve the physical surface cell properties. Silicon treated plants triggers the release of phenolic compounds such as lignin and thus up-regulates CAD. Lignin content is correlated with lodging resistance as it provides rigidity and mechanical support for the culm. Since silicon provides disease resistance, by impeding attacks by pathogen, the cell wall is very much intact. A strong cell wall is the foundation for the growth and development of a healthy and firm plant, thus minimizing occurrence of lodging. Application of silicon is preferred for it provides multitude effect in rice plants in addition to having a higher retention time. However, combination of Si and PBZ should be explored by using other sources of soluble Si with various concentration of PBZ.

## Author contributions

DD and MRI designed the study, discussed the results, read and approved the manuscript. DD performed the research and wrote the manuscript. MRI secured funding and reviewed the manuscript.

### Conflict of interest statement

The authors declare that the research was conducted in the absence of any commercial or financial relationships that could be construed as a potential conflict of interest.
